# Natural Distribution of Parasitoids of Larvae of the Fall Armyworm, *Spodoptera frugiperda*, in Argentina

**DOI:** 10.1673/031.009.2001

**Published:** 2009-05-19

**Authors:** M. Gabriela Murúa, Jaime Molina-Ochoa, Patricio Fidalgo

**Affiliations:** ^a^ Estación Experimental Agroindustrial Obispo Colombres, Sección Zoología Agrícola, CC 9, Las Talitas (T4101XAC), Tucumán, Argentina; ^b^ CONICET; ^c^ Universidad de Colima, Facultad de Ciencias Biológicas y Agropecuarias, Km. 40, autopista Colima-Manzanillo, Tecomán, Colima (28100), México; ^d^ Department of Entomology, University of Nebraska-Lincoln, Lincoln, NE 68583-0816, USA; ^e^ CRILAR (CONICET), entre Ríos y Mendoza s/n, Anillaco (5301), La Rioja, Argentina

**Keywords:** corn, parasitism rate, abundance, diversity indexes, biogeographic regions, *Campoletis grioti*, *Chelonus insularis*, *Archytas marmoratus*, *A*. *incertus*, *Ophion* sp., *Euplectrus platyhypenae*, *Incamyia chilensis*

## Abstract

To develop a better understanding of the natural distribution of the fall armyworm, *Spodoptera frugiperda* (Smith) (Lepidoptera: Noctuidae), and to update the knowledge of the incidence of its complex of parasitoids. *S*. *frugiperda*, samplings in whorl-stage corn were carried out in provinces of Argentina from 1999 to 2003. *S*. *frugiperda* larvae were collected from corn in localities of the provinces of Tucumán, Salta, Jujuy, Santiago del Estero, La Rioja, Córdoba, San Luis, Chaco and Misiones. In each locality 30 corn plants were sampled and only larvae located in those plants were collected. The parasitoids that emerged from *S*. *frugiperda* larvae were identified and counted. The abundance of the parasitoids and the parasitism rate were estimated. The *S*. *frugiperda* parasitoids collected were *Campoletis grioti* (Blanchard) (Hymenoptera: Ichneumonidae), *Chelonus insularis* (Cresson) (Hymenoptera: Braconidae), *Archytas marmoratus* (Townsend) (Diptera Tachinidae) and/or *A*. *incertus* (Macquart), *Ophion* sp. (Hymenoptera: Ichneumonidae), *Euplectrus platyhypenae* Howard (Hymenoptera: Eulophidae), and *Incamyia chilensis* (Aldrich) (Diptera Tachinidae). *C*. *grioti* was the most abundant and frequent during the five-year survey. Similar diversity of parasitoids was obtained in all the provinces, with the exception of *I*. *chilensis* and *E*. *platyhypenae* that were recovered only in the province of Salta. In the Northwestern region, in Tucumán, *C*. *grioti* and species of *Archytas* were the most abundant and frequent parasitoids. On the contrary, in Salta and Jujuy *Ch*. *insularis* was the parasitoid most abundant and frequently recovered. The parasitism rate obtained in Tucumán, Salta and Jujuy provinces were 21.96%, 17.87% and 6.63% respectively with an average of 18.93%. These results demonstrate that hymenopteran and dipteran parasitoids of *S*. *frugiperda* occurred differentially throughout the Argentinian provinces and played an important role on the natural control of the *S*. *frugiperda* larval population.

## Resumen

Con la finalidad de obtener un mejor conocimiento de la distributión natural y actualizar los dates sobre la incidencia del complejo de parasitoides del “cogollero del maíz” *Spodoptera frugiperda* (J. E. Smith) (Lepidoptera: Noctuidae) en Argentina, se realizaron muestreos en diferentes provincias desde 1999 a 2003. Se colectaron larvas en plantas de maíz en diversas localidades de Tucumán, Salta, Jujuy, Santiago del Estero, La Rioja, Córdoba, San Luis, Chaco y Misiones. En cada lugar se monitorearon 30 plantas y se muestrearon solo las larvas presentes en ellas. Se identificaron y contabilizaron los parasitoides nacidos. Se estimó la abundancia y frecuencia de los parasitoides y el porcentaje de parasitismo en cada provincia. Los parasitoides encontrados fueron: *Campoletis grioti* (Blanchard), *Chelonus insularis* (Cresson), *Archytas marmoratus* (Town.) y/o *A*. *incertus* (Macquart), *Ophion* sp., *Euplectrus platyhypenae* Howard, e *Incamyia chilensis* (Blanchard). *C*. *grioti* fue el parasitoide más frecuente y abundante durante los cinco años muestreados. En todas las provincias se encontraron las mismas especies de parasitoides con la exceptión de *I*. *chilensis* y *E*. *platyhypenae* que sólo fueron obtenidos en la provincia de Salta. En las provincias del Noroeste, en Tucumán, los parasitoides más abundantes y frecuentes fueron *C*. *grioti y* *Archytas* spp.; mientras que en Salta y Jujuy el más abundante y frecuente fue *Ch*. *insularis*. El porcentaje de parasitismo total fue 18.93%. Los porcentajes de parasitismo obtenidos fueron de 21.96%, 17.87% y 6.63% para las provincias de Tucumán, Salta y Jujuy, respectivamente. Nuestros resultados muestran que los parasitoides de los Ordenes Hymenoptera y Diptera aparecieron diferencialmente en las distintas provincias de Argentina y juegan un rol importante en el control de las poblaciones del cogollero.

## Introduction

The role and importance of biodiversity in agro-ecosystems is widely recognized. Native insects and pathogens are normal parts of functioning agro-ecosystems and can profoundly influence agricultural structure, species composition, and diversity. The evidence suggests that biodiversity can be used to improve pest management, providing a means of determining the effects of agricultural practices on whole communities or on abundance and dynamics of individual species. However, a major problem in all areas of agriculture is the lack of basic research on distribution, abundance and taxonomy of insect pests and their natural enemies (Altieri 1991; Altieri and Nicholls 2002).

The fall armyworm, *Spodoptera frugiperda* (Smith) (Lepidoptera: Noctuidae), is an important pest on many crops including corn, (*Zea mays*), sorghum (*Sorghum vulgare*), cotton (*Gossypium hirsutum*), and diverse pasture grasses. It is widely distributed in North and South America (Sparks 1979). Given the importance of corn crops in this region, this pest has become one of the most serious problems of the continent. In Northern Argentina, *S. frugiperda* is the most important pest of corn causing loss rates that range from 17% to 72% (Perdiguero et al. 1967). Its control is based mainly on the use of chemical insecticides. Considering the damage caused by this pest, biological control is a highly desirable alternative to insecticides for controlling *S. frugiperda* infestations and the success of any biological control project depends on appropriate biological, ecological, and population studies of the species involved (Miller 1983).

*S. frugiperda* has a diverse complex of natural enemies represented by parasitoids, predators, and pathogens. The value of the parasitoids in reducing larval populations has long been recognized (Pair et al. 1986; Molina-Ochoa et al. 2004). Ashley (1979) and Ashley et al. (1982) cited 21 species for South America, 14 of which are shared with North America. Ashley (1986) reported the geographical distributions and classified the parasitism levels for parasitoids of *S. frugiperda* in North and South America. Molina-Ochoa et al. (2003) reported an inventory of parasitoids and parasites of *S. frugiperda* for the Americas and the Caribbean Basin. In Argentina, twelve hymenopteran and eight dipteran species are known parasitoids on this *S. frugiperda* (Vera et al. 1995; Virla et al. 1999; Berta et al. 2000b; Murúa et al. 2003; Murúa and Virla 2004; Murúa et al. 2006). Most of them were found in samplings made in the province of Tucumán, Argentina, and the incidence and abundance of the different parasitoids on *S. frugiperda* was not studied. As a result, the information on the natural distribution and occurrence of the *S. frugiperda* parasitoids in other regions of Argentina is poor or totally absent.

The aim of the present work was to develop a better understanding of the natural distribution and to update the knowledge of the incidence of the *S. frugiperda* parasitoid complex from different provinces of Argentina.

## Materials and Methods

### Sampling sites

**Figure 1.**
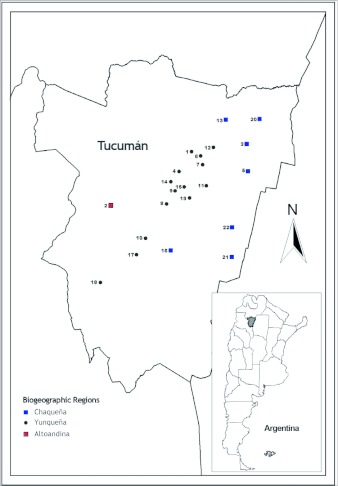
Localities sampled in Tucumán province: 1- El Cadillal, 2- El Mollar, 3- Taruca Pampa, 4- Tafí Viejo, 5- La Virginia, 6- Timbó Viejo, 7- Timbó Nuevo, 8- Lules, 9- El Manantial, 10- Acheral, 11- La Florida, 12- Sunchal, 13- Las Salinas, 14- Yerba Buena, 15- San Miguel de Tucumán, 16- Leales, 17- León Rougués, 18- Monte Bello, 19- San Andrés, 20- El Puesto, 21- El Mojón, 22- Estación Aráoz.

**Figure 2.**
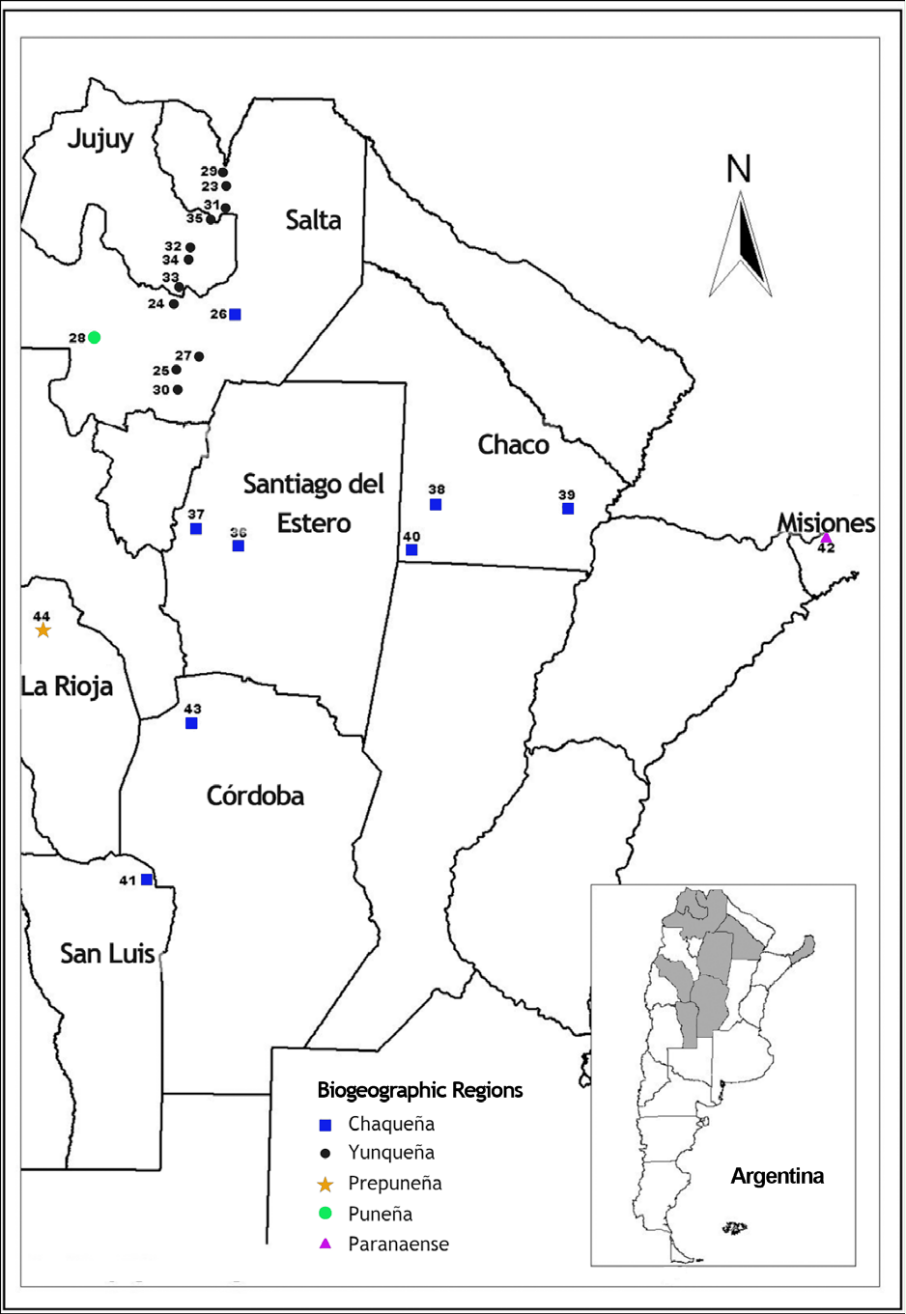
Localities sampled in the provinces of Salta: 23- Orán, 24- Güemes, 25- Metán Viejo, 26- Piquete Cabado, 27- El Galpón, 28- Cachi, 29- Río Pescado, 30- Rosario de la Frontera; Jujuy: 31- Saucelito, 32- Fraile Pintado, 33- Dique Las Maderas, 34- El Quemado, 35- El Bananal; Santiago del Estro: 36- Beltrán, 37- Mula Blanca; Chaco: 38- Charata, 39- Makallé, 40- Gral. Capdevila; San Luis: 41- El Chañar; Misiones: 42- Loreto; Córdoba: 43- Cruz del Eje and La Rioja: 44- Pinchas.

Sampling of *S. frugiperda* larvae was conducted from 1999 to 2003 in different localities of the provinces of Tucumán (22), Salta (8) and Jujuy (5) from the Northwestern region of Argentina (Table 1, 2 and 3) (Figure 1 and 2). Localities were sampled every year when it was possible. Other sporadic samplings were made in the provinces of Santiago del Estero, La Rioja, Córdoba, San Luis, Chaco and Misiones (Table 6) (Figure 2). The results obtained from these samplings were used to study the parasitoids distribution.

### Larval sampling

*S. frugiperda* larvae were collected from whorl-stage corn. In each location 30 corn plants were sampled and only larvae located in those plants were collected. Egg masses and pupae were not collected. Cornfields were monitored all along the cropping season that included early and late planting fields. *S. frugiperda* larvae were placed individually in glass tubes (12 cm high × 1.5 cm diameter), fed with artificial diet (Osores et al. 1982), and held in chambers under controlled conditions at 27 ± 2 °C, 70–75% RH and a photoperiod of 14L: 10D, until emergence of parasitoids.

### Parasitoid identifications

The parasitoids that emerged from larvae were recorded every 24 h. Parasitoids were identified and counted. The dipteran parasitoids, belonging to the family Tachinidae were identified by Susana Ávalos (Facultad de Ciencias Agropecuarias, Universidad Nacional de Cordoba), *Campoletis grioti* (Blanchard) was determined by Carolina Berta (Fundación Miguel Lillo), *Euplectrus platyhypenae* Howard was identified by M. W. Gates and M. Schauff (USDA Systematic Entomology Laboratory, Beltsville, MD), and the remaining parasitoids were identified by the senior author by comparing specimens identified by Luis De Santis (Facultad de Ciencias Naturales y Museo, Universidad Nacional de La Plata).

### Relative abundance and parasitism rate

The number of larvae collected was corrected by subtracting the number that died due to pathogens, parasitic nematodes and/or unknown causes during the first few days after collection before calculating relative abundance and parasitism rate.

**Table I.**
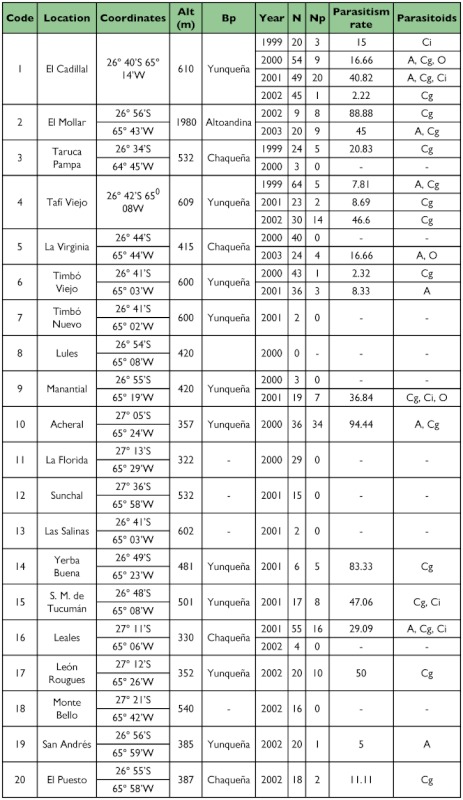
Geographic location, biogeographic province (Bp), sampling year, number of larvae collected (N), number of larvae parasitized (Np), parasitism rate and parasitoid species found in *Spodoptera frugiperda* larvae collected from different localities of the province of Tucumán, Argentina.

**cont:**
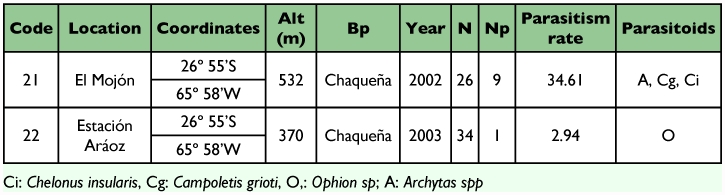

**Table 2.**
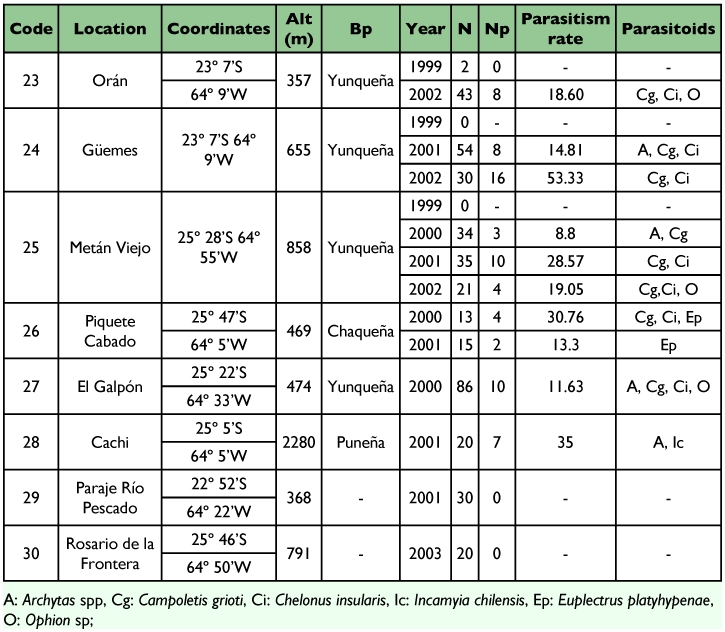
Geographic location, biogeographic province (Bp), sampling year, number of larvae collected (N), number of larvae parasitized (Np), parasitism rate and parasitoids species found in *Spodoptera frugiperda* larvae collected from different localities of the province of Salta, Argentina.

Relative abundance (RA) (Canal Daza 1993; Molina-Ochoa et al. 2001; Molina-Ochoa et al. 2004) was calculated using the following formula: 
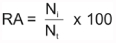



Where the numerator is the number of individuals of species i, and the denominator is the total number of individuals collected.

The parasitism rate (PR) (Van Driesche 1983; Pair et al. 1986; Crisóstomo-Legaspi et al. 2001) was estimated using the following formula: 
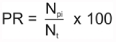



Where the numerator is the number of parasitized individuals of species i, and the denominator is the total number of individuals collected.

### Parasitoids diversity according to different monitored biogeographic regions

To determine if the diversity of S. frugiperda parasitoids was correlated with the environment, the different localities from which parasitoids were obtained were grouped according to the biogeographic region to which they belong. The Shannon - Wiener diversity index (H') (Magurran 1989, Krebs 1995) was estimated for each locality using the following formula:
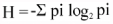



Where pi = relative specific abundance (ni/N), ni = number of individuals of species “i”, and N = number of total individuals collected

**Figure 3.**
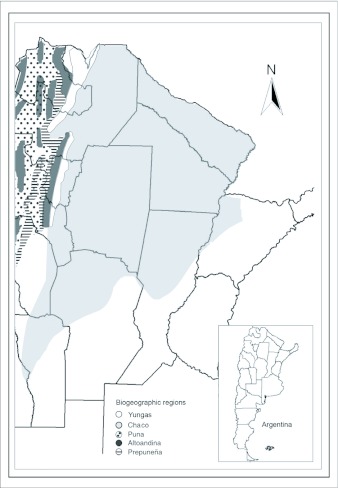
Biogeographic regions from which S. frugiperda larvae with parasitoids were obtained.

A total of five different biogeographic regions were involved in the study (Figure 3). The characteristics of each of them were obtained from Cabrera and Willink (1973) as described below.

### Yunqueña region

The Yungas is an area in the eastern piedmont of the Andes mountains. Its area extends from Venezuela to Northwestern Argentina, between 500 to 2500–3500 m altitude. It is rainy, humid, and warm. The Yungas forests are extremely diverse, ranging from moist lowland forest to evergreen mountain forest and cloud forest. The terrain is extremely rugged and varied, contributing to the ecological diversity and richness. A complex mosaic of habitats occur with changing latitude as well as elevation. There are high levels of biodiversity and species endemism throughout the Yungas regions.

### Chaqueña region

The Chaco is about 647,500 square kilometers in size, and located west of the Paraguay River and east of the Andes, near the Altiplano plateau in Paraguay, Bolivia, and Argentina. It stretches from about 17° to 33° South latitude and between 65° and 60° West longitude. Closer to the mountains in the west, Dry Chaco, is very dry and sparsely vegetated, but going eastward to the Humid Chaco, one encounters thornbrush jungle with ‘quebracho’ trees (*Aspidosperma quebracho-blanco* and *Schinopsis* spp.) and grassy clearings with a wealth of insects. The landscape is mostly flat.

**Table 3.**
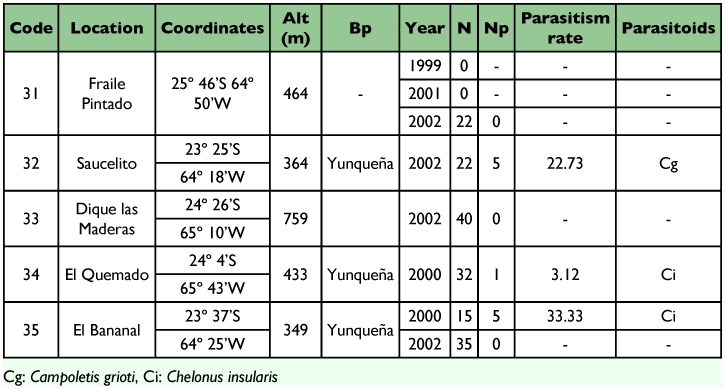
Geographic location, biogeographic province (Bp), sampling year, number of larvae collected (N), number of larvae parasitized (Np), parasitism rate and parasitoids species found in *Spodoptera frugiperda* larvae collected from different localities of the province of Jujuy, Argentina.

### Puna region

The Puna is found above the tree line at 3200–3500 meters elevation, and below the permanent snow line above 4500–5000 meters elevation. It extends from central Peru in the north, across the Altiplano plateau of Peru and Bolivia, and south along the spine of the Andes into northern Argentina and Chile. The flora of the Puna is characterized by alpine bunchgrasses interspersed with herbs, grasses, lichens, mosses, ferns, cushion plants, and occasional low shrubs, with sedges and rushes in poorly drained areas.

### Prepuneña region

The Prepuneña region comprises ravines and dried slopes of Northwest Argentina, from Jujuy to La Rioja provinces, between 1000 and 3400 m altitude. The climate is dry and warm, with summer rain. The vegetation is principally low with scattered shrubs and members of the family Cactaceae.

### Altoandina region

The Altoandina region comprises the upper part of the southern Andes, from latitude 25°S to the tip of the continent in Tierra del Fuego (55°S). This southern part of the Andes includes the highest mountain in the western hemisphere (Aconcagua - 6959 m). The climate is cold and dry, though more humid southward. The scarce precipitation sometimes falls as snow with strong winds. The most important vegetation types are grass-steppe, chamaephyte-steppe and shrub-steppe. Apart from grasses, the grass-steppe sometimes includes mat-forming species.

## Results

Out of 1,652 *S. frugiperda* larvae collected during the five years of surveys in nine provinces of Argentina, 336 produced parasitoids. The parasitoids recovered were: *Campoletis grioti* (Blanchard), and *Ophion* sp. (Hymenoptera: Ichneumonidae); *Chelonus insularis* (Cresson) (Hymenoptera: Braconidae); *Euplectrus platyhypenae* Howard (Hymenoptera Eulophidae); and *Archytas marmoratus* (Townsend) and/or *A. incertus* (Macquart) and *Incamyia chilensis* (Aldrich) (Diptera Tachinidae), (Tables 1, 2, 3 and 4).

### Biology of the different parasitoids found

To increase understanding of the biology and behavior of the different parasitoids collected, and to infer possible causes for the diversity and distribution found, a brief review of their biology is presented.

### Hymenopteran species

#### Campoletis grioti

The genus *Campoletis* Foerster (Hymenoptera: Ichneumonidae) is cosmopolitan and its species are common in habitat surrounded by natural vegetation in moderate climate. Almost all species attack Noctuidae larvae and for this reason it is an important genus in the biological control of different agricultural pests (Porter, 1998; Virla et al. 1999).

*C. grioti* is an oligophagous parasitoid. In Argentina it was found on *S. frugiperda*, *Helicoverpa gelotopoeon*, *Rachiplusia nu* and *Pseudaletia adultera*. In others countries it was cited on *Heliothis virescens*, *Helicoverpa zea* and *Helicoverpa molochitina* (Morey 1971). This species is a solitary larval endoparasitoid and a koinobiont. It attacks the second and third instars larvae. *C. grioti* has three larval instars and when the larva is mature, it leaves its host to start to spin a cocoon and to pupate. The adults are diurnal and live 20 days approximately (Virla et al. 1999; Valverde et al. 1999; Berta et al. 2000a; Murúa 2004).

#### *Ophion* sp

The genus *Ophion* Luteus (Hymenoptera: Ichneumonidae) is cosmopolitan and common in the Neotropical region. Its species live in habitats with natural vegetation and attack Noctuidae larvae, and is an important genus in the biological control of different pests. This genus was found on *H. zea*, *S. frugiperda*, *S. eridania*, *P. adultera*, *Agrotis ipsilon* and *Peridroma saucia* (Porter 1998; Virla et al 1999). All species are solitary larval endoparasitoid and koinobionts. Some species are hyperparasites and attack Braconidae larvae and others Ichneumonidae (Clausen 1940; Virla et al. 1999; Gross and Pair 1991).

#### Chelonus insularis

The genus *Chelonus* Panzer (Hymenoptera: Braconidae) is cosmopolitan, diverse and numerous. In America, 140 species are known. This genus attacks Noctuidae, Gelechiidae and Pyralidae (Marsh 1978; Virla et al. 1999).

**Table 4.**
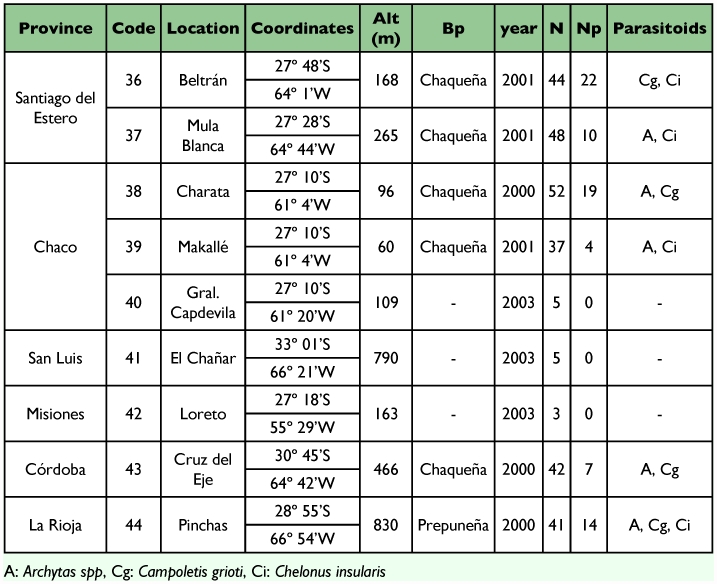
Geographic location, biogeographic province (Bp), sampling year, number of larvae collected (N), number of larvae parasitized (Np), and parasitoids species found in *Spodoptera* *frugiperda* larvae collected in other provinces of Argentina.

*Ch. insularis* is a oligophagous parasitoid of different lepidopteran pests, including *Ephestia elutella*, *Feltia subterranea*, *H. zea*, *Loxostege sticticalis*, *Peridroma saucia*, *S. endania*, *S. exigua*, *S. omithogalli*, *S. praefica*, *S. sunia*, *Trichoplusia ni*, *Elasmopalpus lignosellus*, *Anicla infecta*, *H. virescens* (Medina et al. 1988; Virla et al. 1999). This braconid is considered an excellent candidate for augmentative release, because it can be introduced throughout its overwintering zone, is capable of early-season colonization, and can be used in direct therapeutic releases on target crops (Lewis and Nordlund 1980).

*Ch. insularis* is a solitary, egg-larval endoparasitoid and a koinobiont. The female oviposits in the eggs of the host, but instead of the parasitoid emerging from the eggs they emerge from the immature larvae. There are three larval instars during its life cycle. The third instars larvae emerged from the host larvae, consuming it, and spin a cocoon. Pupation takes place in the foliage and after eight days the adult ecloses and lives approximately 22 days (Ables and Vinson 1981; Medina, et al. 1988; Rezende et al. 1995; Virla et al. 1999; Colomo and Valverde 2002; Murúa 2004).

#### Euplectrus platyhypenae

The genus *Euplectrus* Westwood (Hymenoptera: Eulophidae) is widely distributed throughout the world. Thirty five species have been recorded from the Neotropical region but only five are present in Argentina (De Santis and Esquivel 1966; De Santis 1967, 1979, 1980, 1981, 1989; De Santis and Fidalgo 1994; Virla et al. 1999; Murúa and Virla 2004). All of these species are gregarious ectoparasites of Lepidoptera (Noctuidae and eight other families) and many species are potentially valuable biological control agents (Jones and Sands 1999). Only *E. puttleri* and *E. kuwanae* are monophagous and other species have several hosts (Coudron and Puttier 1988).

*E. platyhypenae* is a koinobiont. Females deposit eggs in clusters on the dorsum of the host, attaching the eggs through a pedicel inserted under the cuticle but above the epidermis. Larval development is completed at the oviposition site. When larvae are ready to pupate, they move down the dead host body and start to spin a cocoon. The emergence of the adult occurs in the morning and they live 15 days (Gonzalez 1985; Murúa and Virla 2004).

### Dipteran species

#### *Archytas* sp

The genus *Archytas* Jaennicke (Diptera: Tachinidae) is cosmopolitan and has 11 species in South America. All species are important in the biological control programs. They attack Noctuidae, Arctiidea, Ctenuchidae, Notodontidae, Pieridae, Geometridae, and Megalopydae (Lepidoptera) larvae (Guimaraes 1977; Virla et al. 1999).

**Table 5.**
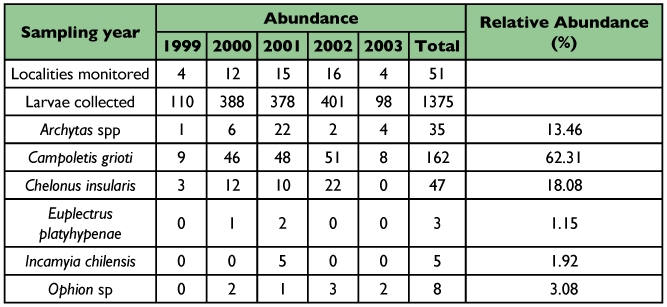
Abundance of larval parasitoids of *Spodoptera frugiperda* collected during five years in the Northwestern provinces of Argentina.

*Archytas marmoratus* and *A. incertus* are solitary larval-pupal parasitoids of numerous species of Noctuidae. Included in their host range are many important pest species in the genera *Helicoverpa*, *Heliothis*, *Pseudaletia*, and *Spodoptera* (Ravlin and Stehr 1984). Females do not oviposit directly on host; instead they deposit numerous eggs in the vicinity of potential host larvae. The eggs soon hatch into planidia-type larvae. Parasitism occurs when a host contacts a planidium that then burrows between the host cuticle and epidermis where it resides (Reitz 1995). In the case of *A. marmoratus*, the first instars feeds on the host larva, but it does not molt until after the host pupates. The first instars must reenter the host following each larval-larval molt of the host. After the host undergoes its larval-pupal molt, the first instars parasitoid penetrates the hemocoel under the host wing pad, where it induces the formation of a respiratory tunnel. *A. marmoratus* development within the host pupa is rapid. The maggot molts to the second instar 1–2 days after host pupation; the second and third instars last 2–4 days each, with pupariation occurring within the host. Because female *A. marmoratus* deposits multiple eggs at one time, and more than one female may oviposit in the same location, considerable potential for superparasitism exists (Reitz 1995).

#### Incamyia chilensis

The genus *Incamya* Townsend (Diptera: Tachinidae) is one of the most common, known and distributed genera of dipteran parasitoids in Chile (Cortés 1968).

*I. chilensis* is polyphagous of different Lepidoptera, principally Noctuidae and Plusiidae families (Caltagirone 1953). *I. chilensis* was found on *S. frugiperda* larvae in Chile, Uruguay and Argentina. This species is a solitary larvalpupal parasitoid. Females deposit numerous eggs near the host larvae. After the third instar the parasitoid larvae leave their host to pupate. The adults eclose after approximately 19 days (Ashley 1979, Caltagirone 1953).

### Relative abundance and parasitism rate

In the Northwestern provinces, *C. grioti* was the most abundant parasitoid followed by *Ch. insularis*, *Archytas* spp., *Ophion* sp., *Incamyia chilensis* and *E. platyhypenae* (Table 5).

The diversity of parasitoids in all provinces was similar. The same parasitoids were obtained except *I. chilensis* and *E. platyhypenae* that were recovered only in the localities of Cachi and Piquete Cabado from Salta province respectively (Tables 2 and 6). In Tucumán the most abundant parasitoids were *C. grioti* and *Archytas* spp.; in Salta and Jujuy, the most abundant was *Ch. insularis* (Table 6).

**Table 6.**
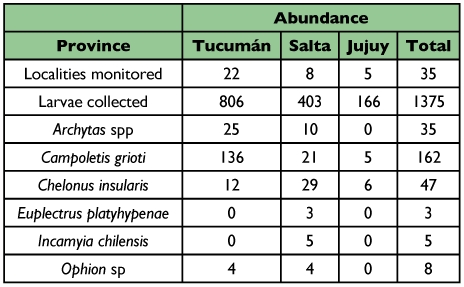
Abundance of larval parasitoids of *Spodoptera frugiperda* collected in three Northwestern provinces of Argentina.

The total parasitism rate during five years was 18.91%. *C. grioti*, *Ch. insularis*, *Archytas* spp, *Ophion* sp., *I. chilensis* and *E. platyhypenae* caused 11.78%, 3.42%, 2.54%, 0.58%, 0.36% and 0.22% of total *S. frugiperda* parasitism, respectively.

The parasitism rate obtained in the province of Tucumán was 21.96% (Table 1). The ichneumonid *C. grioti* caused 16.87%, the species of tachinids caused 3.10%, and *Ophion* sp. and *Ch. insularis* caused 1.36% and 0.49%, respectively. The highest parasitism rate was found in the locality of Acheral in Tucumán (94.44%). Thirty six *S. frugiperda* larvae were collected and 34 produced parasitoids.

**Table 7.**
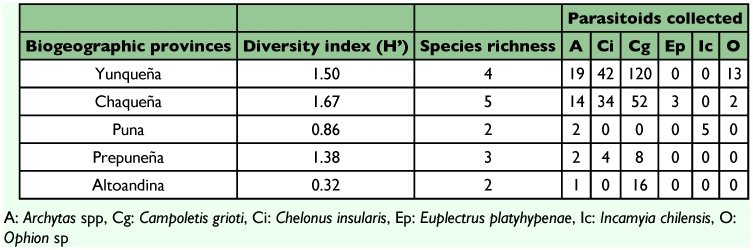
Shannon-Wienner diversity index (H') and species richness found in the different biogeographic provinces.

In the province of Salta (Table 2), the highest rate of parasitism, 53.33%, was obtained in the collection from the locality of Güemes, and it was mainly caused by *Ch. insularis* with about 7.19% of the total parasitism rate in that province. Other species were *C. grioti*, *Archytas* spp., *I. chilensis*, *Ophion* sp., and *E. platyhypenae* with 5.21%, 2.48%, 1.24%, 0.99% and 0.74% parasitism rate, respectively.

In the province of Jujuy (Table 3), the highest parasitism rate was obtained in the locality of El Bananal with 33.33%. The only parasitoids found were *Ch. insularis* and *C. grioti*.

In the provinces of Santiago del Estero, La Rioja and Chaco, the following parasitoids were found: *C. grioti*, *Ch. insularis* and *Archytas* spp, whereas in the province of Cordoba only *C. grioti* and *Archytas* spp were found.

### Parasitoids diversity according to the biogeographic regions

The *S. frugiperda* parasitoid complex showed some similarities but also some differences according to the biogeographic region of the sampling localities. In general, the most abundant parasitoids in all regions were *C. grioti* and *Ch. insularis* followed by *Archytas* spp (Table 7). The highest species diversity obtained was in the Chaqueña region, where five parasitoids were collected, and the diversity of parasitoids in Yunqueña and Chaqueña regions was similar. The same parasitoids were obtained except *E. platyhypenae* which was recovered only in the Chaqueña region (Table 7).

The three most diverse regions were Prepuneña followed by Puna and Altoandina regions (Table 7). In the Puna region the parasitoid collected were only Tachinidae species (*I. chilensis* and *Archytas* spp) and *Ophion* sp.was found in Yunqueña and Chaqueña regions.

## Discussion

The present study evaluated the distribution and parasitism rate of the parasitoids of *Spodoptera frugiperda*, an important pest in corn, from different provinces of Argentina. It updates some previous surveys and it provides new data both in terms of localities evaluated and parasitoid range.

### Relative abundance and parasitism rate

Compared to all the parasitoids previously reported from Argentina (31 species) (Vera et al. 1995; Virla et al. 1999; Berta et al. 2000b; Murúa et al. 2003; Murúa and Virla 2004; Murúa et al. 2006; Murúa 2004) only seven species were recovered in our study. The low diversity of these natural enemies may have been influenced by factors such as insecticides, farming and cultural practices, other natural enemies, and the use of alternative host and climatic conditions.

Kogan et al. (1999) found that cultural practices developed in a plot can affect in a positive or negative way the natural enemy populations, increasing or inhibiting parasitoid colonization in cultivated fields. These practices could also have direct or indirect effects, directly through environment alterations and indirectly affecting the host plant architecture, lack of food, or refuge. The lack of vegetation surrounding the sampling area in cornfields could be another reason for the low diversity found. It is known that the presence of spontaneous vegetation associated with the crop results in a high number and diversity of natural enemies related to this vegetation (Altieri and Whitcomb 1980; Hoballah et al. 2004). In our case a lot of areas were surrounded by lemon groves or soybean crops where insecticide applications are commonly applied. It is also important to consider that *C. grioti*, *Ch. insularis* and *Archytas* spp., that were the more abundant species in this study, are oligophagous parasitoids that attack different hosts of several genera in the family Noctuidae. This may enable them to use alternative hosts in the Northwestern provinces that may allow them to survive insecticides. Odum (1985) mentioned that natural communities have many species with a large number of individuals (common or dominant species) and a lot of species represented by few individuals (uncommon species). A harsh physical environment, contamination or other stress will induce a population decrease of the uncommon species and an increase of the common ones that are best adapted to a variety of stress factors.

Another possible cause for the low diversity is that some samplings were made in cornfields that are planted early in the season when the *S. frugiperda* infestation rate was low (Willink et al. 1993a, b), and as consequence, the number of infested larvae were also low, which reduced the number of parasitoids collected. Conversely, in other collection sites corn wasplanted later and the fields were surrounded by native vegetation that had little anthropogenic disturbance that would affect potential parasitoid refuges.

In northern Argentina, the introduction and adoption of genetically modified (*Bt*) corn hybrids targeted against *S. frugiperda* and other lepidopteran corn pests in the region has increased recently. This technology reduces larval feeding on corn leaves and allows better growth and development of the plant. The concomitant reduction in *S. frugiperda* populations results in an absence of larvae for the parasitoids. Another factor that modulate the parasitoid complex is temperature. Murúa et al (2006) found that temperature was the most important climatic factor influencing parasitoid populations in two localities in Tucumán province. Similar responses have been reported in the Tucumán region for parasitoids attacking the citrus leafminer, *Phyllocnistis citrella* (Diez 2001) and the fruit flies, *Ceratitis capitata* and *Anastrepha fraterculus* (Schliserman 2001).

### Hymenopteran species

*Campoletis grioti* was the most abundant and frequent parasitoid recovered in our study. Similar observations were reported by Murúa et al. (2006) who found that this was one of the most abundant species in Tucumán. Luchini and Almeida (1980) recorded *S. frugiperda* parasitoids occurring in Brazil, and considered *C. grioti* the most important parasitoid. Other authors highlighted the importance of species of *Campoletis* as parasitoids of *S. frugiperda* in different regions of the Americas (Isenhour 1988; Hoballah et al. 2004; Molina-Ochoa et al. 2004). Isenhour (1988) reported that *C. sonorensis* was the most abundant species in Georgia (USA), Hoballah et al. (2004) reported this species as the dominant parasitoid on *S. frugiperda* larvae collected in the State of Veracruz (Mexico), and Molina-Ochoa et al. (2001 (2004) reported the importance of *C. flavicincta* attacking *S. frugiperda* larvae in Nayarit, Jalisco, Colima, and Michoacán in Mexico.

It is important to point out that according to the results of this study, and considering that *C. grioti* was cited occurring in the provinces of Santa Fé and Tucumán (Virla et al. 1999), the distribution of *C. grioti* is extended to the area of Salta Jujuy, Santiago del Estero, La Rioja, Córdoba and Chaco.

The percent parasitism by *C. grioti* found in this investigation was similar to those showed by others authors. Berta et al. (2000a) reported that *C. grioti*., *S. frugiperda* larvae parasitism ranged between 5.26% and 50% in cornfields with and without insecticide application in Tucumán, respectively, and Murúa et al. (2006) working with *S. frugiperda* during four years in Tucumán reported that *C. grioti* was responsible for 39.4% and 5.4% parasitism in two different regions. However, Luchini and Almeida (1980) considered *C. grioti* the most important parasitoid of *S. frugiperda* causing about 95% parasitism.

*Chelonus insulans* was the second most abundant species found in this study. Murúa et al. (2006) also reported that this species is the second most frequently recovered in cornfields in Tucumán, and Virla et al. (1999) determined that this species is very common in that province. The importance of *Ch. insulans* as a *S. frugiperda* parasitoid was emphasized by many authors such as Ashley (1986 Ashley (1988) who listed *Ch. insularis* occurring in Central America and the USA. Pair et al. (1986) working in Southern United States and Northeastern Mexico found that *Ch. insularis* was the most common species. Molina-Ochoa et al. (2003 (2004) reported that *Ch. insularis* had the broadest distribution in Latin America including South America, the Caribbean Basin and the USA and reported that this parasitoid is one of the most abundant natural enemies of *S. frugiperda* larvae in the Western Coast and Gulf of Mexico.

The collections made during this study extended the distribution of *Ch. insularis* (Virla et al. 1999) to the area of Salta, Jujuy, Santiago del Estero, La Rioja and Chaco. The percent of parasitism caused by this braconid in this investigation was similar to that reported by Pantoja and Fuxa (1992), Molina-Ochoa et al. (2001 (2004), Hoballah et al. (2004) and Murúa et al. (2006). The latter authors determined that this species caused 3.30% of total *S. frugiperda* parasitism in cornfields in Tucumán. However the parasitism rate in our tests was generally lower than the values reported by Ashley (1986 Ashley (1988) and Pair et al. (1986), who registered values that ranging between 40 and 70%.

Low rates of occurrence and parasitization of *Ophion* sp. were recorded in this study. Similar observations were reported by Murúa et al. (2006). These authors affirmed that this ichneumonid caused the lowest level of *S. frugiperda* parasitism (0.6%) in Tucumán. In contrast, Gross and Pair (1991) stated that *O. flavidus* (Brullé) caused 19.51% of *S. frugiperda* parasitism in southern Georgia, Rohlfs and Mack (1983) reported that this species parasitized up to 25% of the *S. frugiperda* larvae in Alabama (USA) and Molina-Ochoa et al. (2004), found that *O. flavidus* caused 6.71% parasitism rate in Mexico.

*Euplectrus platyhypenae* was the species with the lowest abundance and frequency in this study. However, Molina-Ochoa et al. (2003 (2004) found that *E. platyhypenae* is one of the most relevant and well distributed parasitoids in tropical Americas and the USA and reported that this species was the most important and widely distributed eulophid parasite of *S. frugiperda* larvae in the Western Coast and Gulf of Mexico. The parasitism rate by this species was similar to that found by Hoballah et al. (2004) and Molina-Ochoa et al. (2001) working with *S. frugiperda* in Mexico. In contrast, Ashley et al. (1980) found that the percentage of parasitism on *S. frugiperda* larvae by this species was 16% in Florida and Pantoja, and Fuxa (1992) in Puerto Rico registered 3.61% of *S. frugiperda* larvae attacked by this species.

### Dipteran species

*Archytas* spp were the third most abundant and common parasitoid in this study. Similar observations were reported by Murúa et al. (2006). The importance of *Archytas* spp. in Argentina and other South American countries have been emphasized by Molina-Ochoa et al. (2003), Molinari and Ávalos (1997), and Virla et al. (1999), who reported that *A. incertus* and *A. marmoratus* were the most prevalent parasitoids in South America as *S. frugiperda* natural enemies. The *S. frugiperda* parasitism rate by *Archytas* spp obtained in this survey was lower than the found by Murúa et al. (2006) in the Tucumán region, but Gross and Pair (1991) found percentage parasitism of *S. frugiperda* by *A. marmoratus* was about 10% in the USA.

*Incamyia chilensis* had the most limited distribution in this survey. Caltagirone (1953) showed that *I. chilensis* is a polyphagous species of different Lepidoptera, principally Noctuidae and Plusiidae families. Ashley (1979) reported that *I. chilensis* was found on *S. frugiperda* larvae in Chile, Uruguay and Argentina.

### Parasitoid diversity according to different monitored biogeographic regions

The differences in climatic conditions, mainly rain regime and temperature, between Yunqueña and Chaqueña regions could be could be a cause for the differences in the parasitoid complexes found. As mentioned before, temperature is one of the most important climatic factor influencing parasitoid populations (Diez 2001; Schliserman 2001; Murúa et al. 2006).

The Chaqueña region is the largest biogeographic region where extensive fields are sown with different crops every year including corn, sunflowers, cotton, and rice. This could be the reason for the presence of *E. platyhypenae* in this region. It is also important to consider that this Eulophidae is an oligophagous parasitoid and attacks different Noctuidae hosts. This species would have other alternative hosts in the Chaqueña region and would be able to survive insecticide applications that are used to control the pests.

The presence of *I. chilensis* in the Puna region coincides with distribution of the parasitoids. This genus is one of the most common in Chile and is widely distributed throughout the Chilean central zone in alfalfa, clover, potato and bean crops (Caltagirone 1953; Cortés 1968).

The genus *Ophion* is found in natural vegetation habitats and attacks a variety of lepidopteran pests. This could be the reason for *Ophion* sp. establishment in the Yunqueña and Chaqueña regions.

### Conclusions

The low diversity of parasitoids found in this investigation can be attributed to different factors such as insecticides, farming and cultural practices that are regularly undertaken in corn fields in Northern Argentina, other natural enemies, alternative host and climatic factors. These results demonstrated that hymenopteran and dipteran parasitoids of *S. frugiperda* occurred at different levels throughout the Argentinian provinces. Moreover, here the distribution of some *S. frugiperda* parasitoids is extended. The diversity analyses of the *S. frugiperda* parasitoid complex showed some differences among biogeographic regions. The ones with extreme climates had lower diversity. In general, the most abundant parasitoids (*C. grioti* and *Ch. Insularis* followed by *Archytas* spp.) were found in all the sampled regions, showing a great capacity to adapt to different environments and hence a great potential for their use as biological control agents. However, the rate of parasitism was variable.
